# Post-Graduate Study in London

**Published:** 1900-10-06

**Authors:** 


### POST-GRADUATE STUDY IN LONDON.

The number of medical men who, although they are qualified for practice, yet wish to continue, or it may be to return to their medical studies, is very considerable, and it has been found worth while by several of the London hospitals to cater specially for them.

Post-graduate students may be roughly divided into two sets. First, there is the young newly-qualified man, often from a provincial or a colonial school, who comes up to London to finish his education; and then there is the older man who after being in practice for some years, perhaps in the country or in foreign lands, or in the army or the navy, wishes to bring himself up to date in all that concerns the practice of his profession.

For the first of these two groups attendance at the hospitals, and more particularly at the special hospitals, is the great attraction which London offers; while for the older group some teaching in modern methods is often a thing to aim at, and they, in addition to attending hospital cliniques, may find it useful to join such special post-graduate classes as those provided by the Polyclinic, the West London Post-Graduate School, or the London School of Tropical Medicine.

In days gone by all the hospitals in London were open to anyone who chose to present his card and ask to go round with any member of the staff; and to a large extent this is still the case, especially if the stranger will arrange not to repeat his visits to the same hospital too often. It is obvious, however, that such desultory attendance, although always interesting and often extremely instructive to the man who only comes up to town for a day or two, are not the thing wanted by the serious post-graduate student who wishes to make the most of his time. For the guidance of the growing number of post-graduate students, then, we give the following list of some of the principal institutions and organisations which they will find useful for their purpose.

### THE SCHOOL HOSPITALS.

Nowhere in the world probably is there such a collection of interesting and instructive cases as are to be found in the great London hospitals, and we have no hesitation in saying that whatever else a post-graduate student may do, unless he comes to London for the study of some single specialty, he must arrange to make steady and repeated visits to the great teaching hospitals of the Metropolis. With the object of organising such visits, of giving the post-graduates a certain position, and of removing them from the category of mere casual visitors, nine of the London medical schools have combined to issue a conjoint ticket which gives the holder the right to attend the clinical instruction, operations, and necropsies at each of the hospitals included in the scheme. The price of this ticket is seven guineas for three months' and ten guineas for six months' attendance.

Full particulars of the arrangements can be obtained on application to the Honorary Secretary, Metropolitan Schools of Medicine, West Wing, Examination Hall of the Conjoint Examining Board, Victoria Embankment, London, W.C., between the hours of 12.30 and 3 p.m., any day except Saturday.

### THE LONDON MEDICAL GRADUATES' COLLEGE AND POLYCLINIC, 22 CHENIES STREET, GOWER STREET, W.C.

This is an institution which has been specially arranged for the use of post-graduates, being intended to serve not merely as a place of instruction but also as a place of union for them.

The building is thoroughly well suited for its purpose, being centrally situated just off Gower Street, and within touch of various means of public conveyance by which hospitals in every part of London may easily be reached.

It contains all the appliances necessary for teaching, including lecture rooms, ophthalmoscope and laryngoscope rooms, Röntgen-ray room, a large laboratory in which practical courses are given on chemical pathology and clinical microscopy, an admirable museum, and a rapidly extending library. There is also a reading-room, open to all members of the college, in which the principal medical journals may be seen.

Lectures are constantly given by a strong teaching staff, the aim being to give to the post-graduate, who does not always care to join a junior class, instruction of such a character as is likely to prove useful to him.

A leading feature of the Polyclinic is the afternoon consultation, either an open consultation or a clinical lecture being given every day except Saturday.

A detailed syllabus of the classes and of the other work of the college may be obtained on application to the

Medical Superintendent, The Polyclinic, 22 Chenies Street, Gower Street, W.C.

It may be added that the classes are so arranged as not to interfere with attendance on the hospitals.

### THE WEST LONDON HOSPITAL.

This hospital is not a very large one, but it is officered by a very active and progressive staff, and in connection with it a systematic scheme for post-graduate teaching has been arranged. The clinical material in its wards and out-patient department is entirely devoted to the instruction of qualified practitioners, and to such also the school appliances, such as lecture rooms, reading and writing rooms, &c., are entirely reserved, it being held by the organisers that there are great advantages in keeping the instruction of students, who always have an examination before them, and post-graduates, who are free from that incubus, entirely separate.

The scheme of study includes instruction in the medical and surgical out-patient rooms; demonstrations in the wards on fixed days; practical instruction in the various special departments, which are here very well organised, and to which clinical assistants are appointed; instruction in the post-mortem room and clinical laboratory; and attendance at operations and at courses of practical lectures.

### CHARING CROSS HOSPITAL.

At this hospital, which is very conveniently situated for the visitor to London, special series of clinical lectures and practical demonstrations, exclusively arranged for practitioners and post-graduate students, are given throughout the year, the lectures being arranged in three courses, each of which consists of ten meetings and lasts ten weeks. Two of these courses are given during the winter session and one during the summer.

### UNIVERSITY COLLEGE.

Classes are held at University College, by the Professor of Pathological Chemistry, which are intended for qualified practitioners and advanced students. The classes are of a practical character, and include analytical work, each student thus learning to apply his knowledge by actual practice.

### KING'S COLLEGE, STRAND, W.C.

So far as post-graduate work is concerned, King's College is best known by the admirable and extremely useful practical course which has for many years been conducted by Professor Crookshank on practical bacteriology. The laboratory is well arranged, every student goes with his own hands through the whole course, and the lectures and demonstrations are most interesting.

### THE LONDON SCHOOL OF TROPICAL MEDICINE, ROYAL VICTORIA AND ALBERT DOCKS, E.

The Seamen's Hospital Society possesses two hospitals, one the Dreadnought Hospital at Greenwich, with 235 beds; and the other the Branch Hospital at the Royal Victoria and Albert Docks, with 50 beds. The society also has two dispensaries, one at Gravesend and the other in the East India Dock Road. This school, although it caters also for senior students, is essentially a post-graduate school, its special function being to afford to practitioners opportunities for becoming familiar with diseases incidental to tropical climates, and for the study of operative surgery. The school has its own special buildings, laboratories, museum, library, &c., which are situated within the grounds of the Branch Hospital. It is open daily, and clinical instruction is given every day in the wards. There are chambers for a few resident students, who must either be already qualified or be in their fifth year. There are several appointments connected with the school, and the Craggs travelling scholarship of £300 is in its gift. Capital opportunities for the study of tropical diseases are offered by this institution.

### NATIONAL HOSPITAL FOR THE PARALYSED AND EPILEPTIC.

In connection with this hospital a very important school of neurology has grown up in an informal sort of way, in consequence of the world-wide fame of many of the members of its medical staff; and although the number of men who regularly attend its practice may not be very great, it is a place to which all medical visitors to London go, and where they can see such a collection of cases of diseases of the nervous system as perhaps is nowhere else to be met with.

### OPHTHALMIC HOSPITALS.

Those who take out the conjoint ticket of the metropolitan hospitals will have no difficulty in finding plenty of opportunities for the study of diseases of the eye at one or other of the hospitals to which they will thus have access, but others who wish to make a special study of this subject will do well to enter at the Royal London Ophthalmic Hospital (the old Moorfields Hospital), City Road, E.C. Students of the hospital are eligible for certain appointments by which they get charge of patients. Other ophthalmic hospitals are: the Royal Westminster Ophthalmic, King William Street, West Strand; the Royal Eye Hospital, St. George's Circus, Southwark, S.E.; and the Central London Ophthalmic Hospital, Gray's Inn Road, W.C.

### GYNÆCOLOGY.

The same remark applies to the study of gynæcology as we have just made in regard to ophthalmology. It can be studied at the general hospitals. Some of the special women's hospitals rather make a point of their not being open to the regular attendance of students. The Hospital for Women in Soho Square, however, is a regular teaching school. It has a large and well-organised clinical department, an extensive out-patient clinique, and it gives a certain number of appointments as clinical assistants, &c.

### THROAT HOSPITALS.

Besides the special departments attached to all the large hospitals, there are several special hospitals for diseases of the throat, at which much can be learned. They are as follows: The Hospital for Diseases of the Throat, Golden Square, W.C.; the Central London Throat Hospital, Gray's Inn Road; the London Throat Hospital, Great Portland Street.

### CHILDREN'S HOSPITALS.

The best known of these is the Hospital for Sick Children in Great Ormond Street, W.C. The East London Hospital for Children is another large institution which offers facilities for post-graduate students; and there are several others where much good work may be done.

### OTHER HOSPITALS.

OTHER hospitals at which cases of interest may be seen are: St. Peter's Hospital for Stone and Urinary Diseases, Henrietta Street, Covent Garden; the Brompton Hospital for Consumption; the North London Hospital for Consumption (open-air treatment); the Queen Charlotte's Hospital (a school of midwifery); the Blackfriars Hospital for Diseases of the Skin and the St. John's Hospital for Diseases of the Skin; the Bethlem Royal Hospital (insanity); the Temperance Hospital (a general hospital treating its patients without alcohol); and the various hospitals of the Metropolitan Asylums Board (fever and smallpox). At most of these arrangements can be made for special study.

